# Translation Elongation Factor 1A Facilitates the Assembly of the Tombusvirus Replicase and Stimulates Minus-Strand Synthesis

**DOI:** 10.1371/journal.ppat.1001175

**Published:** 2010-11-04

**Authors:** Zhenghe Li, Judit Pogany, Steven Tupman, Anthony M. Esposito, Terri Goss Kinzy, Peter D. Nagy

**Affiliations:** 1 Department of Plant Pathology, University of Kentucky, Lexington, Kentucky, United States of America; 2 Department of Molecular Genetics, Microbiology, and Immunology, UMDNJ Robert Wood Johnson Medical School, Piscataway, New Jersey, United States of America; Fundación Instituto Leloir-CONICET, Argentina

## Abstract

Replication of plus-strand RNA viruses depends on host factors that are recruited into viral replicase complexes. Previous studies showed that eukaryotic translation elongation factor (eEF1A) is one of the resident host proteins in the highly purified tombusvirus replicase complex. Using a random library of eEF1A mutants, we identified one mutant that decreased and three mutants that increased *Tomato bushy stunt virus* (TBSV) replication in a yeast model host. Additional *in vitro* assays with whole cell extracts prepared from yeast strains expressing the eEF1A mutants demonstrated several functions for eEF1A in TBSV replication: facilitating the recruitment of the viral RNA template into the replicase complex; the assembly of the viral replicase complex; and enhancement of the minus-strand synthesis by promoting the initiation step. These roles for eEF1A are separate from its canonical role in host and viral protein translation, emphasizing critical functions for this abundant cellular protein during TBSV replication.

## Author Summary

Plus-stranded RNA viruses are important pathogens of plants, animals and humans. They replicate in the infected cells by assembling viral replicase complexes consisting of viral- and host-coded proteins. In this paper, we show that the eukaryotic translation elongation factor (eEF1A), which is one of the resident host proteins in the highly purified tombusvirus replicase complex, is important for *Tomato bushy stunt virus* (TBSV) replication in a yeast model host. Based on a random library of eEF1A mutants, we identified eEF1A mutants that either decreased or increased TBSV replication. *In vitro* studies revealed that eEF1A facilitated the recruitment of the viral RNA template for replication and the assembly of the viral replicase complex, as well as eEF1A enhanced viral RNA synthesis *in vitro*. Altogether, this study demonstrates that eEF1A has several functions during TBSV replication.

## Introduction

Genome-wide screens for host factors affecting RNA virus infections have led to the identification of several hundreds host proteins in recent years [1], [2], [3], [4], [5], [6], [7]. These works demonstrated complex interactions between the host and plus-stranded (+)RNA viruses, the largest group among viruses. (+)RNA viruses contain relatively small genomes and greatly depend on the resources of the infected hosts in many steps during the infection process. These viruses recruit numerous host proteins to facilitate their replication and spread [8], [9], [10]. Many host RNA-binding proteins have been implicated in replication of (+)RNA viruses, including ribosomal proteins, translation factors and RNA-modifying enzymes [8], [9], [10], [11], [12], [13], [14]. In spite of the extensive effort, the actual function of host factors in (+)RNA virus replication is known only for a small number of host factors [8], [10], [15], [16], [17].

*Tomato bushy stunt virus* (TBSV) and other tombusviruses are model plant RNA viruses with 4.8 kb genomic (g)RNA coding for two replication proteins, termed p33 and p92^pol^, and three proteins involved in cell-to-cell movement, encapsidation, and suppression of gene silencing [18], [19]. Yeast (*Saccharomyces cerevisiae*) expressing p33 and p92^pol^ replication proteins can efficiently replicate a short TBSV-derived replicon (rep)RNA [20], [21]. The tombusviral repRNA plays several functions, including serving as a template for replication and as a platform for the assembly of the viral replicase complex [19], [22], [23]. The viral RNA also participates in RNA recombination [6], [18], [24], which likely plays a major role in virus evolution.

One of the major advantages of studying TBSV replication is the availability of genomic and proteomic datasets on virus-host interactions [4], [5], [6], [7], [10], [15], [25], [26], [27]. For example, systematic genome-wide screens of yeast genes have revealed that TBSV repRNA replication is affected by over 100 different host genes [5], [7]. Additional genome-wide screens with TBSV also identified ∼30 host genes affecting TBSV RNA recombination [4], [6], [28]. The identified host genes code for proteins involved in various cellular processes, such as translation, RNA metabolism, protein modifications and intracellular transport or membrane modifications [3], [5], [7].

Additional global approaches based on the yeast proteome microarray (protein array) have led to the identification of over 100 host proteins that interact with viral RNA or the viral replication proteins [25], [26]. Also, proteomics approaches with the highly purified tombusvirus replicase has determined at least seven proteins in the complex, including the viral p33 and p92^pol^, the heat shock protein 70 chaperones (Hsp70, Ssa1/2p in yeast), glyceraldehyde-3-phosphate dehydrogenase (GAPDH, encoded by *TDH2* and *TDH3* in yeast), pyruvate decarboxylase (Pdc1p), Cdc34p ubiquitin conjugating enzyme [14], [26], [27] and eukaryotic translation elongation factor 1A (eEF1A) [25]. The functions of GAPDH and Hsp70 have been studied in some detail [14], [29], [30], [31], but the roles of the other host proteins, such as eEF1A, in the replicase complex are currently undefined.

eEF1A is a highly abundant cellular protein with a role in delivering aminoacyl-tRNA to the elongating ribosome in a GTP-dependent manner. Many additional functions have been ascribed to eEF1A including quality control of newly produced proteins, ubiquitin-dependent protein degradation, and organization of the actin cytoskeleton [32], [33]. Although eEF1A has been shown to be part of replicase complexes of several RNA viruses [16], [34], [35], [36], studies on determining its functions in virus replication are hindered by several major difficulties. These include (i) genetic redundancy: yeast has two eEF1A genes (*TEF1* and *TEF2*), whereas animals and plants have 2–7 genes and several isoforms of eEF1A. (ii) eEF1A provides essential functions for cell viability and mutations could have pleiotropic effects on protein translation, actin bundling and apoptosis. (iii) eEF1A is a very abundant protein that constitutes 1–5% of total cellular proteins, making it difficult to completely remove eEF1A from biochemical assays using cell extracts. (iv) eEF1A is also required for the translation of viral proteins in infected cells, making it difficult to separate its effect on translation versus replication, processes that are interdependent.

The first evidence that translation elongation factors, such as EF-Tu and EF-Ts, play a role in (+)RNA virus replication was obtained with bacteriophage Qbeta [34]. The eukaryotic homolog of EF-Tu, eEF1A was found to bind to many viral RNAs, including the 3′-UTR of *Turnip yellow mosaic virus* (TYMV) [37], West Nile virus (WNV), Dengue virus, *Tobacco mosaic virus* (TMV) and *Turnip mosaic virus* (+)RNA [35], [38], [39], [40]. In addition, eEF1A has also been shown to interact with various viral replication proteins or the replicases, such as the NS5A replication protein of Bovine viral diarrhea virus (BVDV) [41], NS4A of hepatitis C virus (HCV) [42], the TMV replicase [43], and the Gag polyprotein of HIV-1 [44]. It is also part of the replicase complex of vesicular stomatitis virus, a negative-stranded RNA virus [45].

The actual biochemical functions provided by eEF1A for (+)RNA virus replication are currently poorly understood. In case of WNV, eEF1A is co-localized with the WNV replicase in the infected cells and mutations in the WNV (+)RNA within the mapped eEF1A binding site have led to decreased minus-strand synthesis [46]. On the contrary, eEF1A was shown to enhance translation but repressed minus-strand synthesis of TYMV *in vitro* [37], [47], [48]. Overall, eEF1A likely plays a role in the replication of many RNA viruses. The interactions of eEF1A with viral RNAs and viral replication proteins and its high abundance in cells might facilitate recruitment of eEF1A into virus replication.

eEF1A has been shown to interact with the components of the tombusvirus replicase, including the 3′-UTR of the repRNA, as well as the p33 and p92^pol^ replication proteins [25]. eEF1A is also known to interact with the yeast Tdh2p (GAPDH) [49], which is also a component of the tombusvirus replicase. Overall, the multiple interactions of eEF1A with various components of the tombusvirus replicase could be important for eEF1A to regulate yet unknown functions of the viral replicase complex.

In this paper, we characterized the functions of eEF1A in TBSV replication based on identification of functional eEF1A mutants in yeast as well as using *in vitro* approaches. The obtained data support the model that eEF1A plays several roles during TBSV replication, including facilitating the assembly of the viral replicase complex. Moreover, using *in vitro* replication assays, we demonstrate that eEF1A enhances minus-strand synthesis via stimulating the initiation step of the viral RNA-dependent RNA polymerase. Since eEF1A is also associated with several other viral replication proteins or binds to viral RNAs, it is possible that the uncovered functions of eEF1A might be utilized by other RNA viruses during their replication as well.

## Results

### Identification of eEF1A mutants affecting TBSV RNA accumulation

**Figure 1:**
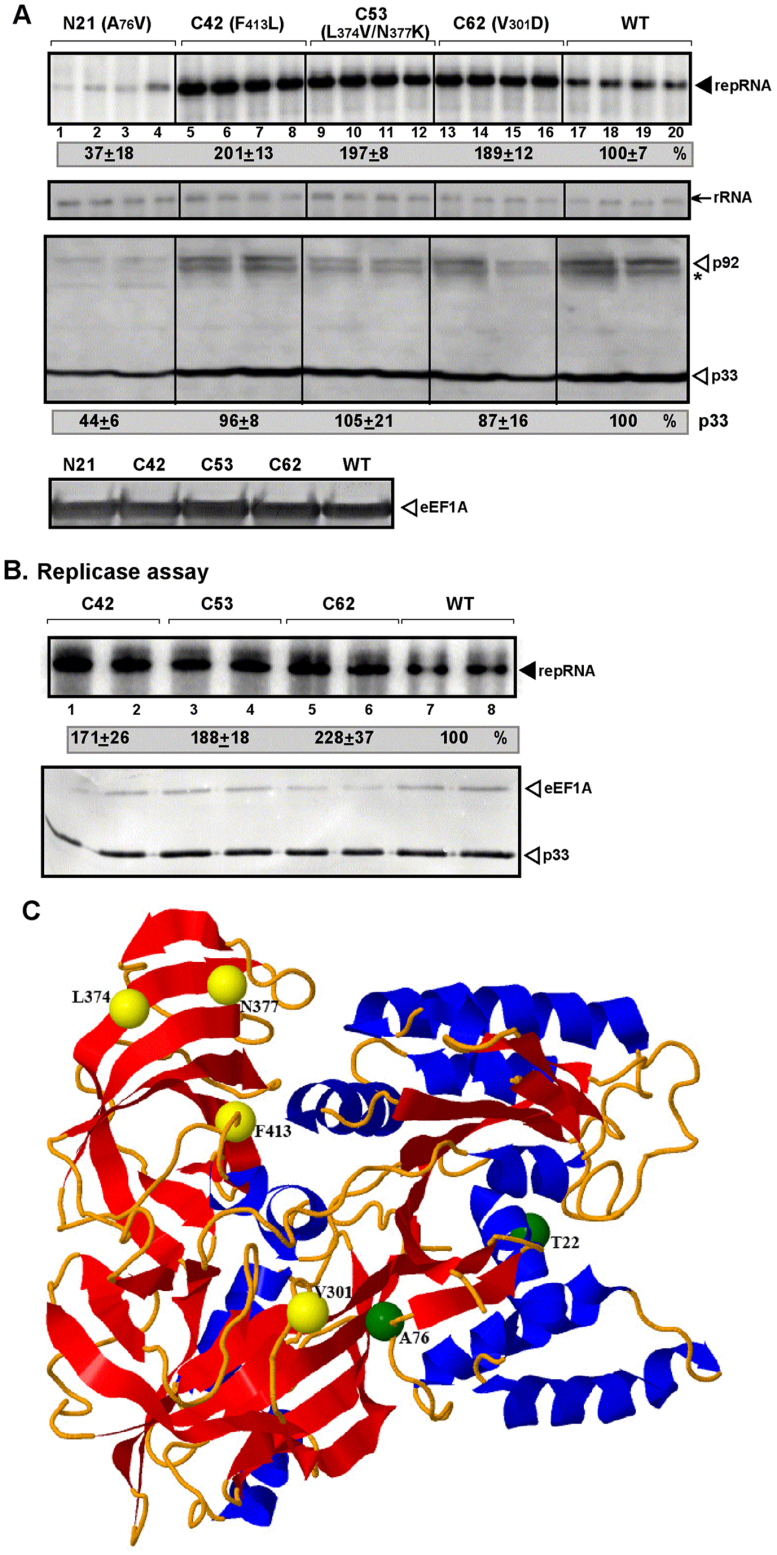
The effect of eEF1A mutations on TBSV repRNA accumulation in yeast. (A) The yeast strains expressed only one form of eEF1A, as indicated. Top panel: Replication of the TBSV repRNA was measured by Northern blotting 24 h after initiation of TBSV replication. The accumulation level of repRNA was normalized based on the rRNA (middle panel, the 18S ribosomal RNA levels were estimated by Northern blotting). Bottom two panels: Accumulation of p33/p92^pol^ and eEF1A was estimated by Western blotting using anti-His and anti-eEF1A antibody, respectively. Note that * marks an SDS-resistant p33 homodimer band. (B) An *in vitro* replicase assay to test the relative activity of the tombusvirus replicase obtained from yeast expressing various mutants of eEF1A. Top panel: We tested the *in vitro* replicase activity using comparable amounts of affinity-purified replicase with added DI-72 RI(−) RNA template. Bottom panels: Western blot analysis showing p33 viral replication protein and the co-purified eEF1A in the above purified replicase preparations. (C) Critical eEF1A residues for tombusvirus replication. Three novel mutants of eEF1A were identified, which exhibited increased tombusvirus replication (V301D, L374V/N377K, and F413L; yellow balls) while the new A76V and the previously identified T22S exhibited decreased tombusvirus replication (green balls). The structure of eEF1A was generated using Jmol with PDB coordinates 1IJE.

To determine the functions of eEF1A during tombusvirus replication, we generated ∼6,000 yeast strains expressing eEF1A with random mutations (see Fig. S1A) and tested the level of TBSV repRNA accumulation in a high-throughput assay [50]. In this assay, we used yeast strains, in which the two wt eEF1A genes (*TEF1/TEF2*) were deleted from the chromosome, while the wt or mutated eEF1A was expressed from plasmids. Importantly, a given eEF1A mutant is the only source of eEF1A in the yeast cells used. Using the high-throughput assay, we identified one yeast strain (N21) expressing an eEF1A mutant that supported reduced TBSV repRNA replication, while the other three strains with eEF1A mutants (named C42, C53 and C62) showed increased level of repRNA accumulation (Fig. 1A and S1B–G). Interestingly, the eEF1A mutants supporting increased steady-state level of repRNA accumulation did not increase the relative level of p33 and p92^pol^ replication proteins (Fig. 1A, bottom panel; S1D–E). Thus, these eEF1A mutants likely affect TBSV replication directly. Accordingly, affinity purification of the solubilized tombusvirus replicase complex from yeast cells, followed by in vitro replicase activity assay revealed that the replicase from C42, C53 and C62 mutant eEF1A-expressing yeast strains had ∼2-fold increased activities when compared with wt eEF1A-expressing yeast strain (Fig. 1B, lanes 1–6 versus 7–8). The amounts of replication protein p33 and the co-purified eEF1A were comparable in the purified replicase samples (Fig. 1B, bottom panel), indicating that the differences in replicase activities in the mutants are likely due to enhanced replicase functions, and not due to altered proteins levels in the replicase complexes. Testing the ability of C42, C53 and C62 mutant eEF1As to bind to the viral RNA or to the p33 and p92^pol^ replication proteins in vitro (Fig. S2B–C) did not reveal significant differences between the mutants and the WT. This further supports that these eEF1A mutations likely increase the function of the viral replicase without altering the protein and RNA components in the replicase.

Placing the identified mutations in the three novel gain-of-function mutants of eEF1A (V_301_D, L_374_V/N_377_K, and F_413_L; Fig. 1C, indicated with yellow balls), which exhibited increased tombusvirus replication, over the known structure of eEF1A [51] revealed a cluster on one face of eEF1A (namely, the actin bundling domain III), away from the domains known to bind to tRNA and translation factor eEF1Bα. On the other hand, the new reduced function mutant (A_76_V, Fig. 1C, indicated with green balls) and the previously identified T_22_S [25], which exhibited decreased tombusvirus replication, showed a distinct and separate localization.

### eEF1A mutants enhance TBSV RNA replication in a cell-free extract

**Figure 2:**
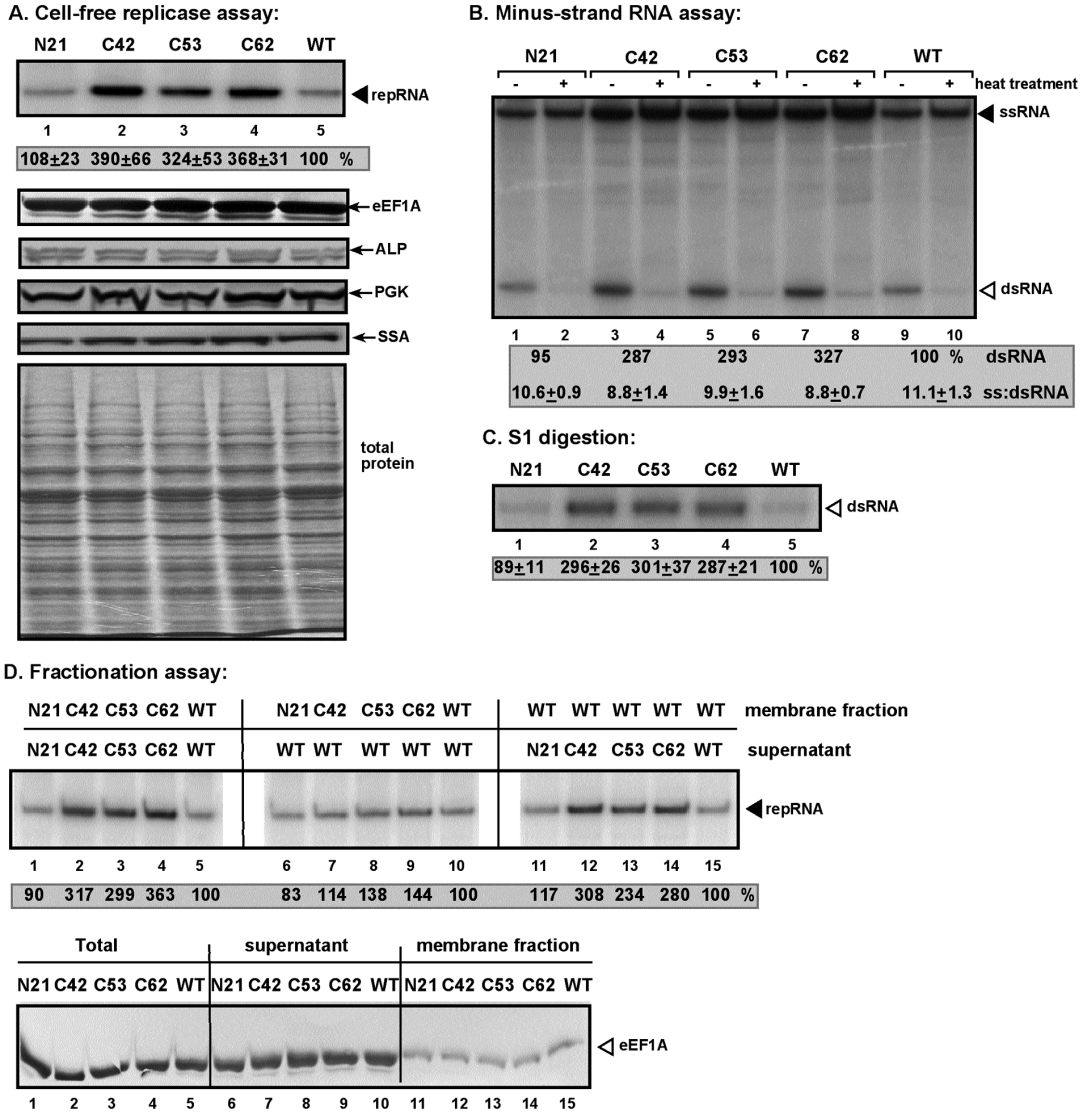
Cell-free TBSV replicase assay supports a role for eEF1A in minus-strand synthesis. (A) Purified recombinant p33 and p92^pol^ replication proteins of TBSV in combination with DI-72 (+)repRNA were added to the whole cell extract prepared from eEF1A mutant or WT yeast strains as shown (lanes 1–5). Top panel: The denaturing PAGE analysis of the ^32^P-labeled repRNA products obtained is shown. The full-length repRNA is pointed at by an arrow. Panels below show Western blot analysis of the whole cell extracts for the indicated yeast proteins based on specific antibodies. Bottom panel shows the coomassie-blue stained SDS-PAGE gel to visualize total protein levels in the whole cell extracts. (B) Detection of single- and double-stranded RNA products produced in the cell-free TBSV replicase assay. Odd numbered lanes represent replicase products, which were not heat treated (thus both ssRNA and dsRNA products are present), while the even numbered lanes show the heat-treated replicase products (mostly ssRNA is present). The amount of dsRNA and the ratio of ssRNA/dsRNA in the samples are shown. Note that, in the nondenatured samples, the dsRNA product represents the annealed (−)RNA and the (+)RNA, while the ssRNA products represents the newly made (+)RNA products. (C) Denaturing PAGE analysis of the TBSV replicase products obtained in the cell-free replicase assay after S1 nuclease treatment, which cleaves the ssRNA, but not the dsRNA product. (D) The denaturing PAGE analysis of the ^32^P-labeled repRNA products obtained in the *in vitro* reconstitution assay is shown. The membrane fraction of the whole cell extracts prepared from eEF1A mutant strains were mixed with the supernatant fraction of CFE prepared from WT eEF1A (lanes 6–10) or the supernatant fraction of CFE from the mutant strains were added to the membrane fraction from the wt strain (lanes 11–15). The reconstituted extracts were programmed with purified recombinant TBSV p33/p92^pol^ and (+)repRNA. Bottom panel: Western blot analysis shows the amount of endogenous eEF1A in various fractions (see above) prepared from yeast expressing various mutants of eEF1A.

Since eEF1A is part of the tombusvirus replicase complex [25], it is possible that C42, C53 and C62 eEF1A mutants might affect the assembly/activity of the tombusvirus replicase. To test this idea, we prepared cell-free extracts (CFE) from yeast strains expressing selected eEF1A mutants in the absence of the wt copy of eEF1A. These yeast extracts contained comparable amount of total proteins as well as the amounts of eEF1A, ALP, PGK and Hsp70 (Ssa) yeast proteins were comparable (Fig. 2A). The advantage of the CFE extracts is that they can then be programmed with the TBSV (+)repRNA in the presence of purified recombinant p33 and p92^pol^ obtained from *E. coli* that leads to the in vitro assembly of the viral replicase, followed by a single cycle of complete TBSV replication, resulting in both (−)-stranded repRNA and (+)-stranded progeny [31], [52]. Therefore, this assay can uncouple the translation of the viral proteins from viral replication, which are interdependent during (+)RNA virus infections.

Using CFEs from yeast expressing one of the three mutant eEF1As resulted in ∼3-fold increased TBSV repRNA accumulation when compared with the extract obtained from yeast expressing the wt copy of eEF1A (Fig. 2A, lanes 2–4 versus 5). These data suggest that the viral replicase complex containing the mutant eEF1A can support *in vitro* TBSV repRNA replication more efficiently than the replicase with the wt eEF1A. In contrast, CFE from N21 yeast supported TBSV repRNA replication to similar extent as the CFE containing wt eEF1A (Fig. 2A, lanes 1 versus 5), indicating that N21 eEF1A mutant can perform the same functions as the wt eEF1A *in vitro*, when the same amount of p33 and p92^pol^ was provided.

To test if the increased TBSV repRNA replication *in vitro* was due to enhanced (+) or (−)-strand synthesis, we analyzed the replication products under non-denaturing versus denaturing conditions (Fig. 2B). These experiments showed that the amount of dsRNA [representing the ^32^P-labeled (−)RNA product hybridized with the (+)RNA] increased ∼3-fold in case of C42, C53 and C62 mutants (lanes 3–8, Fig. 2B) in comparison with the wt (lanes 9–10). The dsRNA nature of these products was confirmed by the ssRNA-specific S1 nuclease digestion assay (Fig. 2C). On the other hand, the ratio of dsRNA and ssRNA did not change in the various CFEs containing the eEF1A mutants or the wt (Fig. 2B). These results are consistent with the model that the replicase complex carrying the eEF1A mutants increased mostly the level of (−)RNA production, which then led to proportionately higher level of (+)RNA progeny.

Cell-fractionation assay, followed by the cell-free TBSV replication assay demonstrated that the soluble fraction from the C42, C53 and C62 mutant yeasts stimulated the *in vitro* replication of TBSV repRNA by ∼3-fold, while the membrane fraction when derived from C42, C53 and C62 mutant yeasts had a lesser effect (Fig. 2D, lanes 12–14 versus 7–9). These data are in agreement with the expected mostly cytosolic distribution of eEF1A, albeit eEF1A is also present in the membrane fraction in a smaller amount (Fig. 2D, bottom panel).

### eEF1A stimulates initiation of (−)RNA synthesis by a viral RdRp in vitro

**Figure 3:**
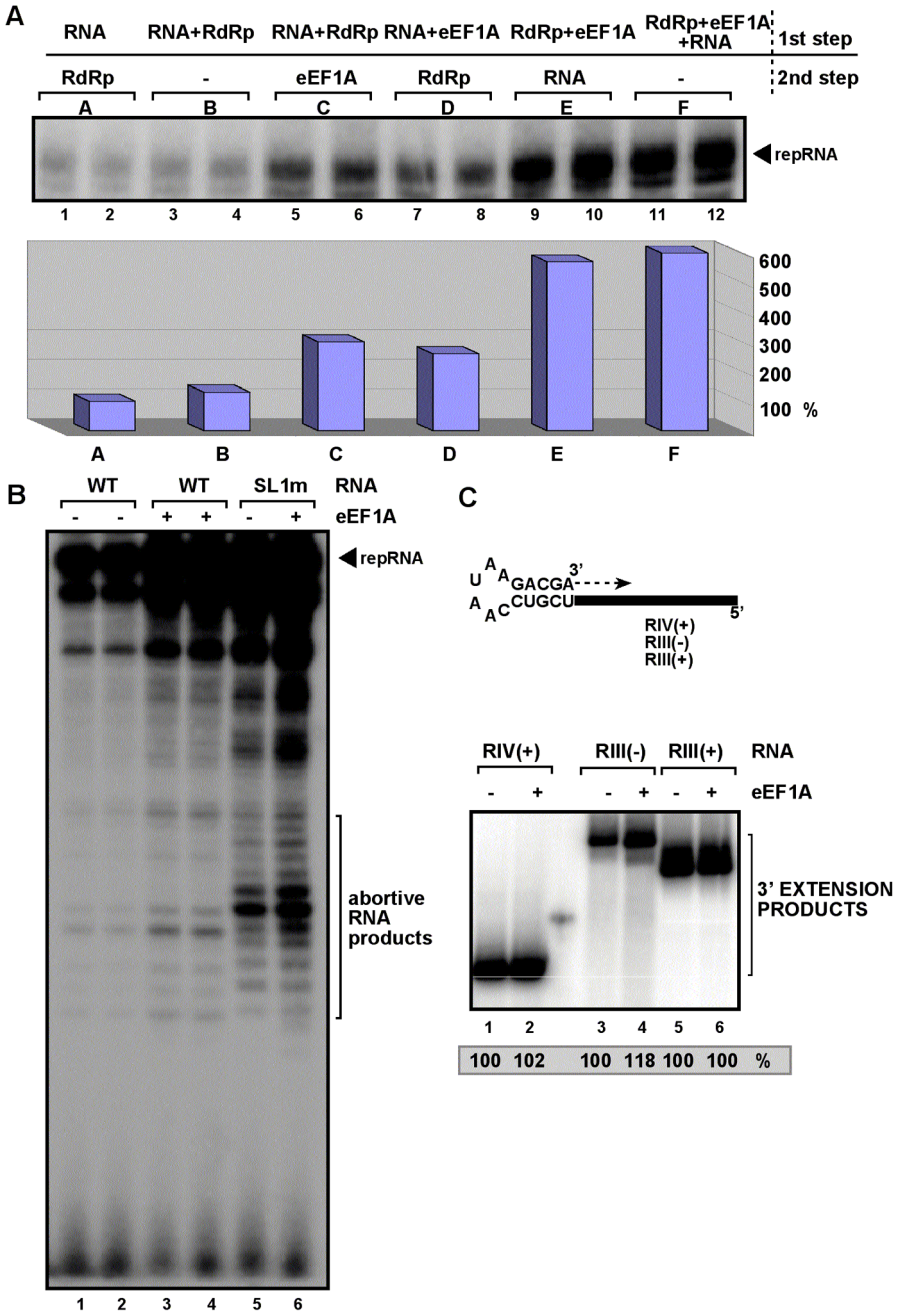
eEF1A promotes the initiation by the TCV RdRp during minus-strand synthesis. (A) Purified eEF1A was added to the TCV RdRp assay as shown. The TBSV (+)RNA template was the short 3′ end region (SL1/SL2/SL3), which contain the promoter region (SL1) for initiation and the replication silencer element (within SL3) that down-regulates initiation. The second template was SL1m with a point mutation within the promoter sequence, which is being used more efficiently by the TCV RdRp *in vitro*. Note that eEF1A has been shown to bind to the replication silencer element. The RdRp assay had two steps: first, the shown components were incubated at room temperature to facilitate their interaction, followed 5 min latter the addition of the shown component and the ribonucleotides to start RNA synthesis. The RdRp activity in samples containing the template RNA and the RdRp were chosen as 100% (lanes 3–4). (B) Detection of abortive RdRp products in the *in vitro* assay. 15% PAGE/UREA gel was used to resolve the 4–10 nt long products produced during initiation followed by rapid termination. Note that abortive RNA products are characteristic products for RNA polymerases that initiate *de novo* (in the absence of a traditional primer). (C) Lack of stimulation of 3′-terminal extension by eEF1A *in vitro*. The template RNAs (shown schematically) contain a common artificial hairpin structure at the 3′ end that facilitates 3′-TEX by the TCV RdRp. The black bar represents 3 different sequences in the three constructs, derived from RIV(+)(includes SL1/SL2/SL3 sequences), RIII(−) and RIII(+) of DI-72 RNA, respectively. The gel image shows the results of 3′-TEX in the presence of 0 or 1 µg eEF1A as shown in a TCV RdRp assay.

To test directly if eEF1A could stimulate RNA synthesis by the viral RdRp, we chose the *E. coli*-expressed recombinant p88^pol^ RdRp protein of *Turnip crinkle virus* (TCV), which is unlike the *E. coli*-expressed TBSV or CNV p92^pol^ RdRp, does not need the yeast cell-free extract to be functional *in vitro* [53], [54]. The template specificity of the recombinant TCV RdRp with TBSV RNAs is similar to the closely-related tombusvirus replicase obtained from yeast or infected plants [21], [54], [55], [56]. However, the recombinant TCV RdRp preparation lacks co-purified eEF1A, unlike the yeast or plant-derived tombusvirus replicase preparations, facilitating studies on the role of eEF1A on the template activity of a viral RdRp. When we added the highly purified wt eEF1A to the RdRp assay containing TCV RdRp protein and a TBSV derived (+)RNA template, which is used by the TCV RdRp *in vitro* to produce the complementary (−)RNA product (Fig. 3A, lanes 3–4) [55], we observed a ∼6-fold increase in (−)RNA synthesis by the TCV RdRp (lanes 11–12), while, as expected, we did not detect new (+)RNA progeny (not shown). This suggests that eEF1A can greatly stimulate TCV RdRp activity *in vitro*, confirming a direct role for eEF1A in (−)RNA synthesis by a viral RdRp.

Since it is known that eEF1A can bind to the 3′-UTR of TBSV (+)RNA as well as to the tombusvirus replication proteins [25] and to the TCV RdRp (Fig. S2A), we wanted to test if the above stimulating activity of eEF1A in the *in vitro* RdRp assay was due to binding of eEF1A to the (+)RNA template and/or to the TCV RdRp protein. Pre-incubation of the purified wt eEF1A with the TCV RdRp prior to the RdRp assay led to a ∼5-fold increase in *in vitro* (−)RNA synthesis (Fig. 3A, lanes 9–10), while pre-incubation of the purified eEF1A with the TBSV (+)RNA template prior to the RdRp assay led only to a ∼2-fold increase in (−)RNA products (lanes 7–8). Also, pre-incubation of the TCV RdRp with the (+)RNA template prior to the RdRp assay containing purified eEF1A led only to a ∼2-fold increase in (−)RNA synthesis (lanes 5–6), suggesting that eEF1A can stimulate (−)RNA synthesis less efficiently after the formation of the (+)RNA-RdRp complex. Overall, data shown in Fig. 3 imply that eEF1A stimulates (−)RNA synthesis most efficiently when it forms a complex with the viral RdRp prior to binding of the template RNA to the eEF1A-RdRp complex.

To test if eEF1A stimulates the rate of initiation of (−)RNA synthesis, we analyzed the amount of abortive RNA products, which are generated during *de novo* initiation of RNA synthesis by the TCV RdRp [57]. We found that the amount of the 5–11 nt long abortive RNA products increased by 3.5-fold in the presence of purified eEF1A in the TCV RdRp assay (Fig. 3B, lanes 3–4 versus 1–2). We also tested the RdRp activity in the presence of eEF1A using a (+)RNA template with a mutation opening the closed structure in the promoter region that leads to increased template activity [58]. The mutated template also showed 2-fold increased abortive RNA products in the RdRp assay with eEF1A (Fig. 3B, lanes 5 versus 6). These data strongly support the model that eEF1A stimulates the *de novo* initiation step in the RdRp assay.

To test if eEF1A stimulates the rate of RNA synthesis in the absence of *de novo* initiation, we analyzed the amount of 3′-terminal extension (3′-TEX) RNA products, which are generated from an internal primer by the TCV RdRp (Fig. 3D) [55]. Addition of purified eEF1A did not increase the amount of 3′TEX products (lanes 2, 4, 6, Fig. 3D), suggesting that the elongation step during complementary RNA synthesis is not affected by eEF1A. Altogether, the obtained *in vitro* TCV RdRp data suggest that eEF1A can mostly stimulate the initiation step during *de novo* viral (−)RNA synthesis.

### Inhibition of replicase activity and template recruitment by inhibitors of eEF1A *in vitro*

**Figure 4:**
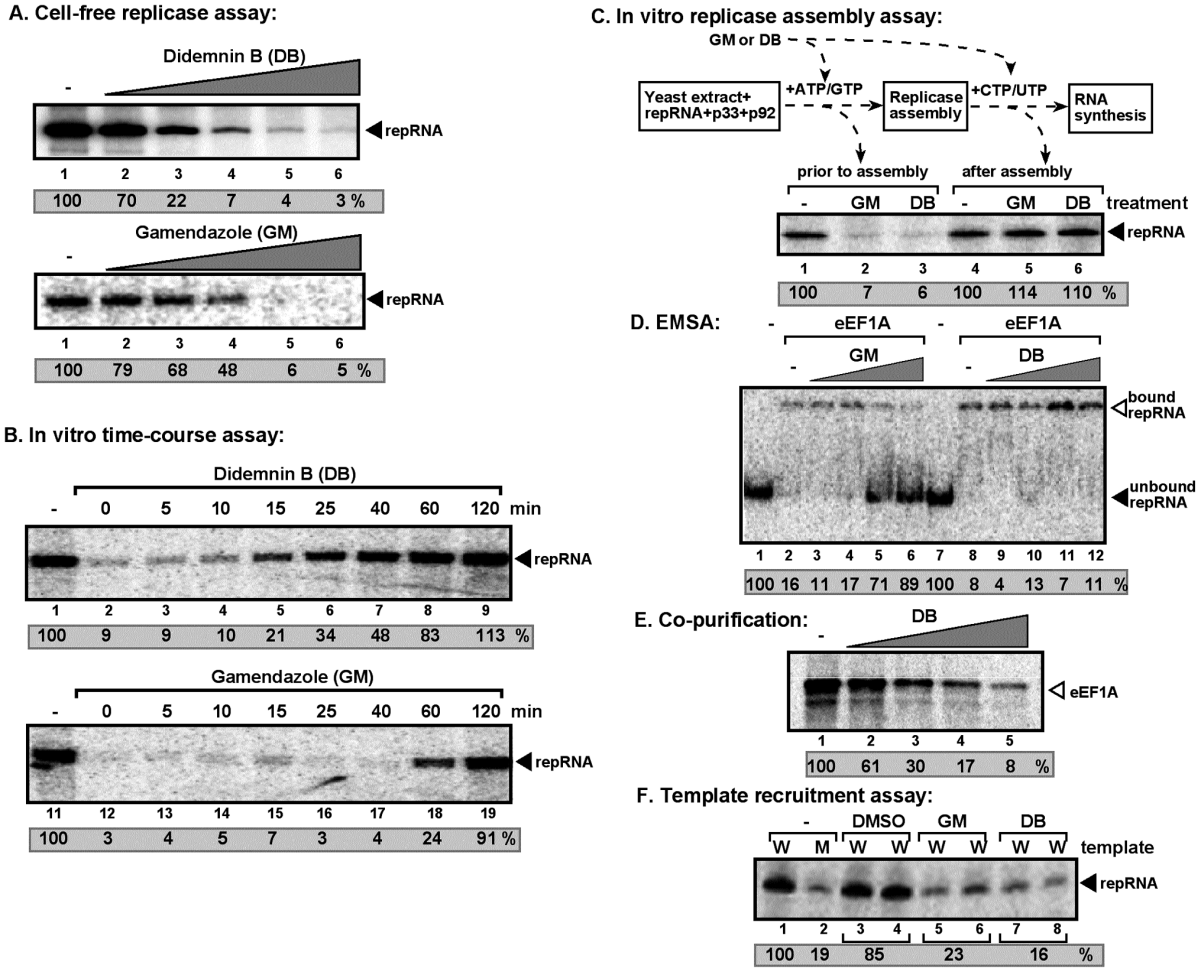
Inhibition of TBSV repRNA replication by Didemnin B and Gamendazole in a cell-free TBSV replicase assay. (A) The cell-free TBSV replicase assay was performed as described in Fig. 2. DB and GM were added in the following amounts: 0, 50, 100, 150, 200, 250 µM for DB and 0, 5, 25, 50, 100, and 200 µM for GM, respectively. The replicase activity in the samples containing the DMSO solvent instead of DB or GM was chosen as 100%. (B) Time course analysis was performed in a cell-free TBSV replicase assay as described in Fig. 2. DB (150 µM) and GM (100 µM) were added at various time points and the replicase assay was stopped after 3 hours for each treatment, followed by RNA analysis in a denaturing PAGE gel. The replicase activity in the samples containing DMSO added at the 0 time point was chosen as 100%. (C) A step-wise approach was used to separate the possible effect of DB and GM during either the assembly of the TBSV replicase or RNA synthesis steps. In step 1, the purified recombinant TBSV p33, p92^pol^ and (+)repRNA were added to the whole cell extract in the presence of ATP and GTP, which only supports the assembly of the TBSV replicase, but prevents RNA synthesis. This was followed by removal of the extra amount of p33, p92^pol^ and repRNA, which were not bound to the membranes of cell-free extract, and then by the standard replicase assay in a buffer containing ^32^P-UTP and ATP, CTP and GTP (step “RNA synthesis”). The denaturing PAGE analysis of the ^32^P-labeled repRNA products obtained is shown. Note that DB (150 µM) and GM (100 µM) were added to the assay either at the beginning (prior to replicase assembly) or after the replicase assembly. See further details in panel B. (D) The effect of DB and GM on binding between the purified eEF1A and ^32^P-labeled template RNA (SL1/SL2/SL3) based on EMSA. The bound and unbound RNAs are pointed at by arrowheads. GM and DB were applied in the following amounts: 0, 5, 50, 250, and 1000 µM. Note that the amount of unbound RNA in the absence of eEF1A (lane 1) was chosen as 100%. (E) The inhibitory effect of DB on co-purification of eEF1A with the viral repRNA. WT ^35^S-labeled eEF1A was produced in a translation assay using rabbit reticulocyte lysate, followed by incubation with biotin-labeled DI-72(+) repRNA in the presence of 0, 50, 150, 500 and 1000 µM DB. Then the repRNA was captured with streptavidin-coated magnetic beads, followed by elution of the co-purified proteins from the beads. SDS-PAGE analysis shows the amount of co-purified ^35^S-labeled eEF1A. (F) The inhibitory effect of DB and GM on the template recruitment step *in vitro*. Purified recombinant p33/p92 and ^32^P-labeled DI-72 (+)repRNA (indicated as W, lanes 1 and 3–8) or C_99_-G mutant (+)repRNA (indicated as M, lane 2) were added to a whole cell extract (CFE) in the presence of DMSO (control), 100 µM GM or 150 µM DB, followed by centrifugation/washing to remove the ^32^P-labeled repRNA that is not bound to the membrane. Then the membrane-bound RNA was analyzed in a denaturing PAGE gel. Note that the recruitment deficient C_99_-G mutant repRNA bound to the membrane nonspecifically (∼20% level).

To further test the function of eEF1A in TBSV replication, we used chemical inhibitors of eEF1A, including Didemnin B (DB) and Gamendazole (GM). DB inhibits the activity of GTP-bound eEF1A during translation by binding to a pocket in eEF1A involved in the interaction with the aminoacylated tRNA and the nucleotide exchange factor eEF1Balpha [59], [60]. GM has been shown to inhibit the actin bundling function, while it does not inhibit protein translation or GTP binding functions of eEF1A [61]. We found that both DB and GM efficiently inhibited TBSV repRNA replication in the in vitro assay with CFE, which contains the endogenous eEF1A (Fig. 4A). Time-course experiments revealed that the inhibition by DB was the most effective when the inhibitor was added at the beginning or during the first 10–15 min of the assay (Fig. 4B, lanes 2–5), while GM inhibited the cell-free replication of TBSV repRNA when added not only at the beginning, but up to 40 min after the start of the assay (lanes 12–17). It is known that the recruitment of the viral RNA and replication proteins as well as the assembly of the viral replicase complex take place during the first 40–60 min in the cell-free assay [31]. Since DB could inhibit translation, we also tested the effect of another translation inhibitor, namely cycloheximide, which did not affect TBSV repRNA replication in our assay (Fig. S3). These data suggest that the inhibition by DB and GM is unlikely through decreased translation in the replication assay. Therefore, the above data are consistent with the model that DB and GM interfere with the assembly of the viral replicase complex in the CFE. Also, GM seems to be a more potent inhibitor of TBSV replication than DB.

To further test if DB and GM can interfere with the assembly of the tombusvirus replicase complex, we performed a two-step *in vitro* assembly/replication assay, also based on CFE containing endogenous eEF1A [31]. In this assay, first, we only provide ATP and GTP in addition to the replication proteins, the (+)repRNA and CFE, which can support the assembly of the replicase, but cannot perform RNA synthesis due to the lack of CTP and UTP [31]. After 1 hr incubation, once the replicase assembly had taken place, we collected the membrane fraction of the CFE by centrifugation and removed the supernatant containing the unbound p33, p92, repRNA as well as the cytosolic fraction of the CFE. Then we added all four rNTPs (including ^32^P-labeled UTP) to the membrane fraction of the CFE to allow for RNA synthesis by the pre-assembled replicase complex (second step, Fig. 4C) [31]. Interestingly, adding either DB or GM during the first step resulted in robust inhibition of TBSV repRNA synthesis during the second step of the assay (Fig. 4C, lanes 2–3 versus 1), whereas providing the same amount of DB and GM at the beginning of the second step did not result in inhibition of repRNA replication (lanes 4–6). These data support a model that DB and GM could inhibit the assembly of the tombusvirus replicase complex, but not the RNA synthesis by the already assembled replicase. Similarly, DB and GM failed to inhibit TBSV RNA synthesis in an *in vitro* assay with a highly purified RdRp from yeast (Fig. S4A).

Since the assembly of the tombusvirus replicase also depends on events prior to the replicase assembly step, such as template RNA binding by the viral replication proteins/host proteins (such as eEF1A), and template recruitment to intracellular membranes [21], [52], we also tested the effect of DB and GM on these processes as well based on purified recombinant eEF1A. We found that GM strongly interfered with the binding of eEF1A to the viral RNA in an EMSA assay (Fig. 4D, lanes 3–6 versus 2), whereas DB did not affect the binding under the assay conditions (lanes 9–12). Since DB binds only weakly to eEF1A in solution, but it binds much more effectively to eEF1A in the presence of GTP and the ribosome [62], we also performed in vitro co-purification experiments. First, ^35^S-labeled eEF1A was produced in an in vitro translation system (containing ribosome and GTP) and, second, biotin-labeled viral (+)repRNA was added. After short incubation in the absence or presence of various amount of DB, we performed affinity-purification of the viral RNA. Phosphoimaging revealed that eEF1A was co-purified with the viral RNA and the amount of protein co-purified with the viral (+)repRNA was inhibited by increasing amount of DB in the assay (Fig. 4E, compare lane 1 with 2–5). This demonstrated that DB inhibits the binding of eEF1A to the viral repRNA. Moreover, both DB and GM interfered with the recruitment of the viral template RNA to the membrane of the CFE containing endogenous eEF1A (Fig. 4F, lanes 5–8 versus 3–4). On the other hand, DB and GM do not seem to affect the interaction between eEF1A and p33 or p92 replication proteins in vitro (Fig. S4B). Altogether, these data suggest that inhibition of eEF1A function by DB and GM could block several steps during the assembly of the tombusvirus replicase complex, including template binding by eEF1A and viral RNA recruitment into replication.

## Discussion

(+)RNA virus replicases contain viral- and host-coded components, which likely provide many yet undefined functions to facilitate robust virus replication in infected cells. Translation factors, such as eEF1A, are among the most common host factors recruited for (+)RNA virus replication. eEF1A is an integral component of several viral replicases, including the highly purified tombusvirus replicase complex. Since eEF1A is an essential G protein involved in translation elongation, it is difficult to obtain evidence for its direct involvement in virus replication in living cells. Indeed, down-regulation of eEF1A in cells has led not only to decreased TBSV repRNA accumulation, but also reduced p33 levels [25]. However, using a small set of functional eEF1A mutants defective in various functions revealed that eEF1A is involved in stabilization of p33 replication protein in yeast [25].

Based on the previous successful strategy of analyzing eEF1A mutants, here we generated ∼6,000 random mutants covering the entire eEF1A sequence and found four mutants, which greatly affected TBSV repRNA accumulation in yeast (Fig. 1A). Among these mutants, C42, C53 and C62 increased TBSV repRNA replication. Importantly, this effect by the eEF1A mutants was not due to changing the translation efficiency of p33/p92^pol^, but likely via directly altering viral replication and affecting the activity of the viral replicase. On the other hand, N21 mutant of eEF1A resulted in decreased TBSV RNA accumulation and also led to reduction in the level of p33 replication protein. This is reminiscent of the previously characterized GDP-binding mutant T_22_S [25], which supported greatly reduced level of viral RNA replication and p33 accumulation due to shortened half-life of p33. Overall, N21 mutant further supports that one of the functions of eEF1A in TBSV replication is to stabilize the p33 replication protein in yeast.

In addition to this genetic evidence on the relevance of eEF1A in TBSV replication in yeast, we also obtained additional supporting data by showing that chemical inhibitors of eEF1A, such as DB and GM, strongly inhibited replication of TBSV repRNA in the cell-free replication assay (Fig. 4A). Since we used the same amount of purified recombinant p33/p92^pol^ in this *in vitro* assay (i.e., translation in the CFE is not needed for production of p33/p92^pol^), the role of eEF1A in TBSV replication must be separate from its role in protein translation. Altogether, these data strongly established that eEF1A is directly involved in TBSV replication, independent of the role of eEF1A in protein translation.

### eEF1A selectively enhances minus-strand synthesis during TBSV replication

The identified eEF1A mutants were also useful to dissect the functions of eEF1A in TBSV replication. Based on a cell-free TBSV replication assay in CFE prepared from yeast expressing the C42, C53 or C62 mutants, we found that the minus-strand synthesis was enhanced by ∼3-fold, while the rate of plus-strand synthesis was proportionate with (−)RNA synthesis, resulting in ∼10-fold more (+) than (−)RNA products for wt and each mutant.

**Figure 5:**
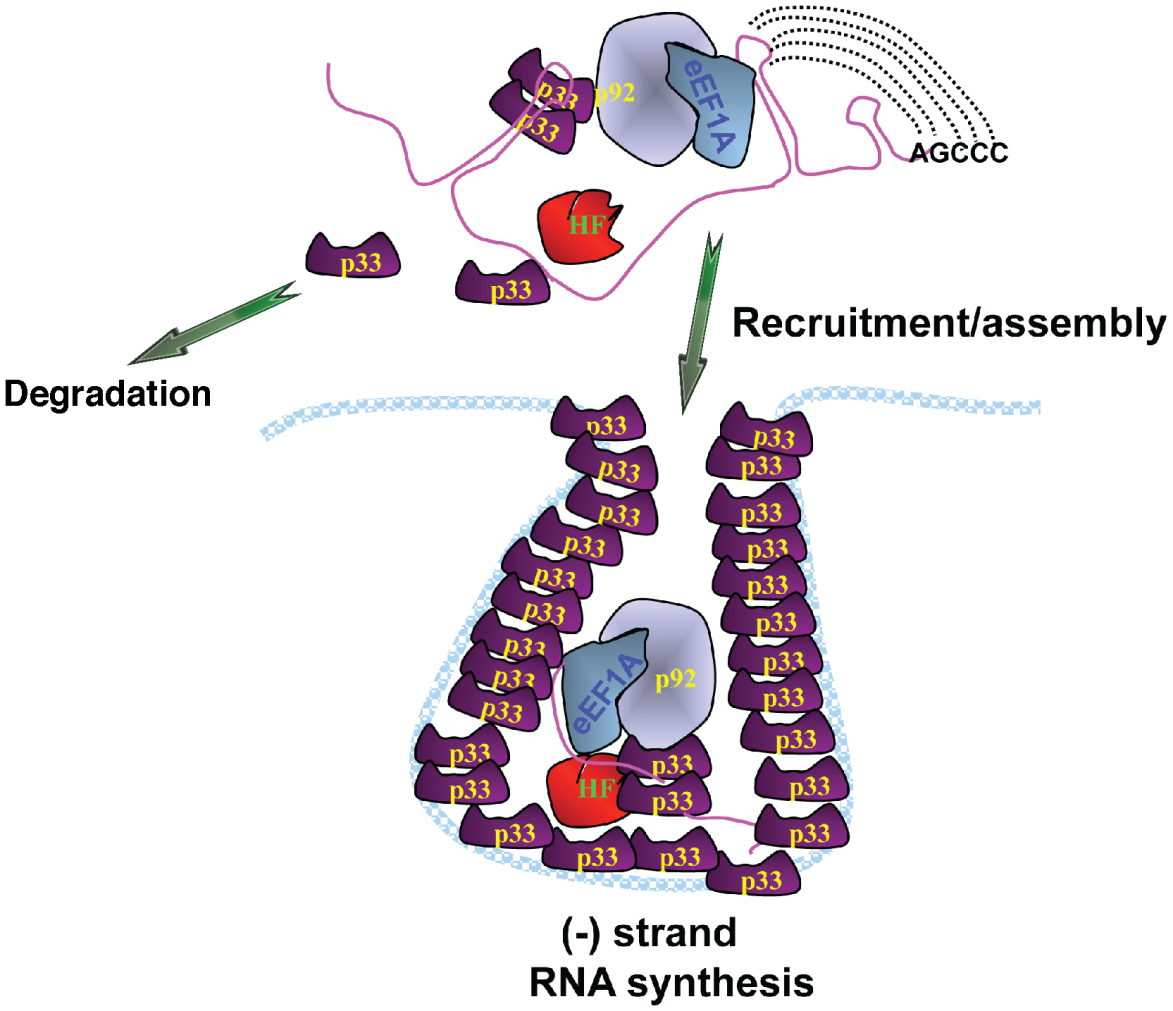
A model describing the functions of eEF1A during tombusvirus replication. eEF1A not only affects the stability of p33 in cells, but it binds to both the p92^pol^ replication protein and the viral RNA, facilitating RNA recruitment into replication and the assembly of the viral replicase complex (shown as a membrane-bound complex with multiple p33, p92^pol^ and additional host factors, HF, such as Hsp70 and Cdc34p). Subsequently, eEF1A promotes minus-strand synthesis by facilitating initiation on the viral template RNA by the viral replicase.

We confirmed a direct role for eEF1A in RNA synthesis *in vitro* by using a highly purified eEF1A and the recombinant TCV RdRp, which is closely homologous with the TBSV p92^pol^. Interestingly, it seems that eEF1A stimulates the RdRp activity directly, since pre-incubation of eEF1A and the RdRp prior to the RdRp assay led to the highest level of stimulation of (−)RNA synthesis (Fig. 3A). On the other hand, pre-incubation of eEF1A with the TBSV-derived template RNA led only to ∼2-fold increase in RNA synthesis *in vitro* (Fig. 3A). Analyzing the amount of short abortive RdRp products, which are produced through initiation followed quickly by abortive termination [57], in the *in vitro* assays revealed that eEF1A strongly enhanced the initiation of minus-strand synthesis (Fig. 3B). Although the actual mechanism of stimulation of RdRp activity by eEF1A is currently unknown, we propose that eEF1A might facilitate the proper and efficient binding of the RdRp to the 3′ terminal sequence of the viral RNA prior to initiation of (−)-strand synthesis (Fig. 5). Accordingly, eEF1A was shown to bind to the so-called replication silencer sequence (RSE) in the 3′-UTR, which is required for the assembly of the viral replicase complex [22], [58]. The binding of eEF1A-RdRp complex to the RSE might assist in placing the RdRp over the 3′-terminal promoter sequence, thus facilitating the initiation of (−)RNA synthesis starting from the 3′-terminal cytosine. Similar function of eEF1A in stimulation of (−)RNA synthesis has been proposed for WNV, based on mutations in the viral RNA within the eEF1A binding sequence that reduced the binding affinity of RNA to eEF1A and inhibited (−)RNA synthesis in infected cells [46].

### eEF1A stimulates the assembly of the viral replicase complex during TBSV replication

Recent intensive work revealed that the assembly of the viral replicase complex is a regulated process involving viral- and host factors, cellular membranes and the viral (+)RNA [8], [10], [19], [63], [64], [65]. The assembly of the viral replicase also depends on steps occurring prior to the actual assembly process, such as selection of the viral template RNA and the recruitment of (+)RNA/protein factors to the sites of assembly. Although our current understanding is rather poor about the factors involved and their functions during replicase assembly, rapid progress is being made in this area due to the development of a new cell-free assay based on yeast CFE [30], [31]. The yeast CFE is capable of assembling the tombusvirus replicase complex *in vitro* in 40–60 min in the presence of recombinant p33/p92^pol^ and the viral (+)repRNA [31], allowing for studies on direct roles of various factors. We find that inhibition of eEF1A activity by either DB or GM also inhibited the assembly of the tombusviral replicase complex based on time-course experiments (Fig. 4B) as well as a direct replicase assembly assay (Fig. 4C). On the contrary, the replicase activity was not inhibited by these compounds after the assembly took place (Fig. 4B–C). It is possible that after the formation of the eEF1A-RdRp-repRNA complex DB or GM are not effective in inhibiting the stimulatory effect of eEF1A on the RNA synthesis by the viral RdRp. Additional *in vitro* experiments with purified tombusvirus replicase preparations confirmed the lack of inhibition of RNA synthesis by DB or GM (Fig. S4A) on pre-assembled virus replicases.

The inhibition of the tombusvirus replicase complex by DB or GM might come from the ability of these compounds to inhibit the template RNA recruitment step (Fig. 4F). If the recruitment of the viral (+)RNA is inhibited, then the assembly of the viral replicase cannot take place in yeast or *in vitro* [21], [22], [31]. A target for GM and DB could be the inhibition of binding between eEF1A and the viral (+)RNA (Fig. 4D, E). Since the actual steps during the replicase assembly process are not yet known, it is possible that eEF1A might play additional roles in the assembly of the viral replicase complex.

The presented data are also in agreement with the function of eEF1A as a chaperone of the viral RdRp. Binding between the eEF1A and RdRp might alter the structure of the RdRp that favors *de novo* initiation for RNA synthesis. Indeed, the chaperone activity of eEF1A and its bacterial homolog EF-Tu has been shown before [66], [67]. Moreover, the EF-Tu-EF-Ts complex is thought to function in the Qbeta replicase complex as a chaperone for maintaining the active conformation of the RdRp protein [68].

Overall, the current work demonstrates two major functions for eEF1A in TBSV replication (Fig. 5): (i) stimulation of the assembly of the viral replicase complex, likely by facilitating the recruitment of the viral RNA template into the replicase; and (ii) enhancement of the minus-strand synthesis by promoting the initiation step. These roles for eEF1A are separate from its canonical role in host and viral protein translation.

## Materials and Methods

### Yeast strains and expression plasmids

*Saccharomyces cerevisiae* strain BY4741 (*MAT* **a** *his3*Δ*1 leu2*Δ*0 met15*Δ*0 ura3*Δ*0*) was obtained from Open Biosystems (Huntsville, AL, USA). Plasmid-borne *TEF1/2* TKY strains (*MATα ura3-52 leu2-3, 112 trp1-Δ1 lys2-20 met2-1 his4-713 tef1::LEU2 tef2Δ pTEF2 URA3*) were published before [69], [70], [71], [72]. The plasmid pESCHIS4-ADH-His33/CUP1-DI-72 expressing *Cucumber necrosis virus* (CNV) p33 and the TBSV replicon RNA, called DI-72, was described earlier [25]. The *LYS2*-based plasmid pRS317-Tet-His92, expressing CNV p92 under the control of Tetracycline-regulatable (Tet) promoter was constructed as follows: the Tet promoter sequence was obtained from pCM189-His92/Tet [73] by digestion with *EcoRI* and *BamHI*, and CNV p92 coding sequences from pGAD-His92 [7] digested with *BamHI* and *PstI*, followed by ligation into pRS317 vector treated with *EcoRI* and *PstI*. To generate mutations within *TEF1* coding sequence by random mutagenesis, we constructed the *TRP1*-based plasmid pRS314-pTEF1-TEF1, which expressed *TEF1* under the control of its native promoter. The *TEF1* promoter sequence, the *TEF1* coding region and the *Cyc1* terminator sequences were amplified by PCR with the following primer pairs, #2764 (CCGCGAGCTCATAGCTTCAAAATGTTTCTAC)/#2765 (CCGCGGATCCGTAATTAAAACTTAGATTAGATTGC), #2768 (CCGCGGATCCAAAATGGGTAAAGAGAAGTCTC)/#1877 (CCGCCTCGAGTTATTTCTTAGCAGCCTTTTGAGCAGC), and #2769 CCGCCTCGAGGAGGGCCGCATCATGTAA/#2770 (CCGCGGTACCAGCTTGCAAATTAAAGCCTTC), respectively. This was followed by cloning the PCR products into pRS314 digested with *SacI* and *KpnI*.

### Random mutagenesis of *TEF1*

The mutagenic PCR conditions were as follows: 50 mM KCl, 10 mM Tris (pH 8.3 at 25°C), 7 mM MgCl_2_, 0.3 mM MnCl_2_, 1 mM dCTP and dTTP each, 0.2 mM dGTP and dATP each, 0.2 µM of each primer, 20 pM of template DNA and 10 units of Taq polymerase in a 10 µl reaction volume in 10 aliquots. The PCR was performed for 30 cycles at 94°C for 1 min, 50°C for 1 min, and 72°C for 1 min in a conventional thermal cycler. Three overlapping ∼300–500 bp N-, central- and C-terminal segments of the *TEF1* gene were amplified separately by PCR using primer pairs: #2767 (GTTTCAGTTTCATTTTTCTTGTTC)/#2788 (GAGTCCATCTTGTTGACAG), #2787 (CATCAAGAACATGATTACTGGTAC)/#2790 (GACGTTACCTCTTCTGATTTC) and #2789 (CGGTGTCATCAAGCCAGGT/#2771, (TTCGGTTAGAGCGGATGTGG), respectively.

### Yeast transformation, plasmid shuffling and replication screening of mutant yeast strains

Yeast strain TKY102 was co-transformed with constructs pESCHIS4-ADH-His33/CUP1-DI-72 and pRS317-Tet-His92 to induce TBSV repRNA replication according to standard Lithium acetate-PEG protocol [74]. The transformed yeast cultures were grown in a Synthetic Complete (SC) media with 2% glucose lacking leucine, histidine, lysine and uracil (SC-ULHK^−^) by shaking at 29°C overnight. To completely suppress TBSV replication before induction, 1 mg/ml Doxycycline was added to the media to inhibit the expression of p92. The plasmid pool carrying the randomly mutated *TEF1* gene was introduced into the yeast cells already transformed with the two virus expression plasmids by *in vivo* gap repair mechanism via homologous recombination (Fig. S1A) [75]. Briefly, pRS314-pTEF1-TEF1 was digested with enzymes to truncate the *TEF1* coding sequence, and then the digested plasmid was recovered. The gapped plasmid (5–10 µg) was transformed together with overlapping PCR (20 µg) products carrying the *TEF1* mutations created by random mutagenic PCR (see above). The transformed yeast cells were selected on SC media lacking uracil, tryptophan, leucine, histidine and lysine. The colonies were further streaked onto SC media plate lacking tryptophan, leucine, histidine and lysine (SC-TLHK^−^) with 0.1% (w/v final) 5-Fluoroorotic Acid (5-FOA) media to select against the *URA3*-based wild-type *TEF1* plasmid (Fig. S1A). This selection was repeated once and the loss of *URA3* plasmid was confirmed by the inability of the yeast strains to grow on uracil-minus media. The yeast cells carrying the randomly mutated *TEF1* were grown at 29°C for 24 h in SC-TLHK^−^ media with 50 µM CuSO_4_ to induce virus replication. Total RNA extraction from yeast cells and Northern blotting and Western blotting were done as previously described [7], [25].

### Replication assay using the whole cell extract

Whole cell yeast extract capable of supporting TBSV replication *in vitro* was prepared as described [31]. The *in vitro* TBSV replication assays were performed in 20-µl total volume containing 2 µl of whole cell extract, 0.5 µg DI-72 (+)repRNA transcript, 400 ng purified MBP-p33, 100 ng purified MBP-p92^pol^ (both recombinant proteins were purified from *E. coli*), 30 mM HEPES-KOH, pH 7.4, 150 mM potassium acetate, 5 mM magnesium acetate, 0.13 M sorbitol, 0.4 µl actinomycin D (5 mg/ml), 2 µl of 150 mM creatine phosphate, 0.2 µl of 10 mg/ml creatine kinase, 0.2 µl of RNase inhibitor, 0.2 µl of 1 M dithiothreitol (DTT), 2 µl of 10 mM ATP, CTP, and GTP and 0.25 mM UTP and 0.1 µl of [^32^P]UTP [31]. The reaction mixture was incubated at 25°C for 3 h. The reaction was terminated by adding 100 µl stop buffer (1% sodium dodecyl sulfate [SDS] and 0.05 M EDTA, pH 8.0), followed by phenol-chloroform extraction, isopropanol-ammonium acetate precipitation, and a washing step with 70% ethanol as described [52]. The newly synthesized ^32^P-labeled RNA products were separated by electrophoresis in a 5% polyacrylamide gel (PAGE) containing 0.5× Tris-borate-EDTA (TBE) buffer with 8 M urea. To detect the double-stranded RNA (dsRNA) in the cell-free replication assay, the ^32^P-labeled RNA samples were divided into two aliquotes: one half was loaded onto the gel without heat treatment in the presence of 25% formamide, while the other half was heat denatured at 85°C for 5 min in the presence of 50% formamide [31]. S1 nuclease digestion to remove single-stranded ^32^P-labeled RNA was performed at 37°C for 30 min in a buffer containing 5 mM sodium acetate (pH 4.5 at 25°C), 0.28 M NaCl, 4.5mM ZnSO4 and 40 U S1 nuclease (Boehringer).

Fractionation of the whole cell extract was done according to [52]. The total extract was centrifuged at 21,000× g at 4°C for 10 min to separate the “soluble” (supernatant) and “membrane” (pellet) fraction. The pellet was re-suspended and washed with buffer A (30 mM HEPES-KOH pH 7.4, 150 mM potassium acetate, and 5 mM magnesium acetate) followed by centrifugation at 21,000× g at 4°C for 10 min and re-suspension of the pellet in buffer A. *In vitro* TBSV replication in the fractions was performed as described [31].

### Protein purification from *E. coli* and yeast

Expression and purification of the recombinant TBSV p33 and p92 and TCV p88C replication proteins from *E. coli* were carried out as described earlier with modifications [54]. Briefly, the expression plasmids were transformed separately into *E. coli* strain BL21 Rosetta (DE3). Protein expression was induced using isopropyl β-D-thiogalactopyranoside (IPTG) for 8 h at 16°C, then the cells were collected by centrifugation (5,000 rpm for 5 min). The recombinant TCV p88C protein was purified on an amylose resin column (NEB), as described [54]. The cells were suspended and sonicated in MBP column buffer containing 20 mM Tris-Cl pH 8.0, 150 mM NaCl, 1 mM EDTA, 10 mM β-mercaptoethanol and 1 mM phenylmethylsulfonyl fluoride (PMSF). The sonicated extract was then centrifuged at 27,000 g for 10 min, followed by incubation with amylose resin (NEB) for 1 h at 4°C. After washing the resin 3 times with the column buffer and once with a low salt column buffer (25 mM NaCl), the proteins were eluted with a low salt column buffer containing 0.18% (V/W) maltose and 6% (V/V) glycerol and stored at −80°C. MBP-p33 and MBP-p92^pol^ were purified as above, except 30 mM HEPES-KOH pH 7.4 was used instead of 20 mM Tris-Cl pH 8.0. eEF1A was purified from yeast as described [76] and stored in aliquots at the vapor temperature of liquid nitrogen. Protein fractions used for the replication assays were 95% pure, as determined by SDS-PAGE.

### Replicase purification from yeast and in vitro RdRp assay

Yeast strains (WT, C42, C53, C62) were transformed with plasmids pESCHIS4-ADH-HF33/CUP1-DI-72 expressing 6XHis- and Flag-tagged CNV p33 and the TBSV DI-72 repRNA, and pRS317-Tet-His92, expressing CNV p92 under the control of Tet promoter [25]. Co-purification was done according to a previously described procedure with the following modification [25]. Briefly, 200 mg of yeast cells were resuspended and homogenized in TG buffer [50 mM Tris–HCl [pH 7.5], 10% glycerol, 15 mM MgCl_2_, 10 mM KCl, 0.5 M NaCl, 0.1% Nonidet P-40 (NP-40), and 1% [V/V] yeast protease inhibitor cocktail (Ypic)] by glass beads using FastPrep Homogenizer (MP Biomedicals). The yeast cell lysate was cleared by centrifugation at 500× g for 5 min at 4°C to remove unbroken cells and debris. The membrane fraction containing the viral replicase complex was collected by centrifugation at 21,000× g for 15 min at 4°C and then solubilized in 1 ml TG buffer with a buffer containing 1% NP-40, 5% SB3–10 [caprylyl sulfobetaine] (Sigma), 1% [V/V] Ypic via gentle rotation for 1 h min at 4°C. The solubilized membrane fraction was centrifuged at 21,000× g for 15 min at 4°C and the supernatant was incubated with 20 µl anti-FLAG M2-agarose affinity resin (Sigma) pre-equilibrated with 0.7 ml TG buffer. After 2 h of gentle rotation at 4°C, we washed the resin 5 times with TG buffer containing 1% NP-40, the resin-bound replicase complex was eluted in 100 µl elution buffer [50 mM Tris–HCl [pH 7.5], 10% glycerol, 15 mM MgCl_2_, 10 mM KCl, 0.05 M NaCl, 0.5% Nonidet P-40 (NP-40), 1% Ypic and 0.15 mg/ml Flag peptide (sigma)]. In vitro RdRp activity assay was performed by using DI-72 RI(−) RNA template transcribed in vitro by T7 transcription [25].

### Gel mobility shift assay (EMSA) and co-purification of eEF1A-repRNA

EMSA was performed in a 10 µl-reaction containing 20 mM HEPES [pH 7.6], 50 mM KCl, 2 mM MgCl_2_, 1 mM DTT, 0.1 mM EDTA, 10% [vol/vol] glycerol, 10 U of RNase inhibitor, 10 nM ^32^P-labeled DI-72 (+) RNA probe and 0.5 µg purified eEF1A protein [76]. Reactions were incubated at room temperature for 20 min and then resolved by 4% nondenaturing polyacrylamide gel as described previously [25].

For in vitro eEF1A-repRNA co-purification, DI-72(+) repRNA was biotin-labeled in standard T7 transcription reaction in the presence of 20 µM Biotin-16-UTP (Roche). After the T7 transcription, the unincorporated biotin-UTP was removed on a Bio-Rad mini gel filtration column. The biotinylated RNA was immobilized on a column containing Streptavidin MagneSphere Paramagnetic Particles (SA-PMPs). Briefly, a 30-µl suspension of SA-PMPs (Promega) was washed three times with 1 ml of water and re-suspended in 1× Phosphate Buffered Saline (PBS). Biotinylated DI-72(+) RNA (5 µg) was then added to the suspension of SA-PMPs, followed by 30 min incubation at 4°C with gentle rotation. The SA-PMPs were collected on the side of the tube in a magnetic stand and washed 3 times with 1× PBS buffer. eEF1A was translated in vitro and labeled with ^35^S methionine using Rabbit Reticulocyte Lysate (Promega) according to manufacturer's manual. The in vitro eEF1A translation product (10 µl) was pre-incubated in a 200 µl binding buffer (20 mM HEPES [pH 7.6], 50 mM KCl, 2 mM MgCl_2_, 1 mM DTT, 1 mM GTP, 0.1 mM EDTA, 10% [V/V] glycerol, 1% BSA, 10 U of RNase inhibitor and 0.2% NP-40) with 150 µM Didemnin B (final concentration) or DMSO for 30 min at 30°C and then incubated with biotinylated DI-72(+) RNA-bound SA-PMPs for 1 h at 4°C. The SA-PMPs were collected in a magnetic stand and washed 5 times with the binding buffer, followed by elution with 30 µl SDS-PAGE sample buffer. The eluted protein samples were resolved by SDS-PAGE and then exposed to phosphorimager.

### *In vitro* TCV p88C RdRp assay

The TCV RdRp reactions were carried out as previously described for 2 h at 25°C [54]. Briefly, the RdRp reactions were performed in a 20 µl reaction containing 50 mM Tris-HCl (pH 8.2), 10 mM MgCl_2_, 10 mM DTT, 1.0 mM each ATP, CTP, and GTP, 0.01 mM UTP plus 0.1 µl of [^32^P]UTP, 7 pmol template RNA, 2 pmol affinity-purified MBP-p88C. 20 pmol eEF1A was added to the reaction at the beginning or as indicated in the text and Fig. 3 legend. The ^32^P-labeled RNA products were analyzed by electrophoresis in a 5% or 15% PAGE/8 M urea gel [57]. The 86-nt 3′ noncoding region of TBSV genomic RNA was used as the template in the RdRp assay [25], [54].

### The use of eEF1A inhibitors in the *in vitro* replicase assembly assay

Purified Didemnin B (NSC 325319) was kindly provided by the Natural Products Branch, NCI (Bethesda, MD, USA), while Gamendazole was a generous gift from Dr. Tash (University of Kansas Medical Center). Both chemicals were dissolved in DMSO (the final concentration was 20 mM). The concentrations of chemical and time point of the addition of the chemicals to the *in vitro* reaction are indicated in the text. The cell-free TBSV replicase assay and the *in vitro* TBSV replicase assembly assay were performed according to [31]. Briefly, the purified recombinant TBSV p33, p92^pol^ and (+) repRNA were added to the cell-free reaction in the presence of 1.0 mM ATP and GTP in step 1. After incubation at 25°C for 1 h, the *in vitro* reactions were centrifuged 21,000× g at 4°C for 10 min. The supernatant containing extra p33, p92^pol^ and repRNA, which were not bound to the membranes in the cell-free extract, was discarded, while the membrane pellet was re-suspended in a standard *in vitro* replicase assay buffer containing [^32^P]-UTP and ATP, CTP, and GTP, and incubated at 25°C for 3 h [31].

### *In vitro* viral RNA recruitment assay

The TBSV viral RNA gets recruited to the membrane from the soluble fraction with the help of TBSV replication proteins and host factors present in the yeast CFE. The *in vitro* RNA recruitment reaction was performed according to [31], except that ^32^P-labeled DI-72 (+)repRNA were used and rCTP, rUTP, ^32^P-labeled UTP, and Actinomycin D were omitted from the reaction. As a negative control, a recruitment-deficient repRNA, termed C_99_-G mutant, was used (Fig. 4F, lane 2) [23]. This mutant RNA is not recognized by p33/p92 replication proteins and it does not replicate in plants, in yeast or in the CFE in vitro [23], [31], [52], [77]. The RNA recruitment assay results in the assembly of the functional viral replicase, when wt repRNA is used, and nonfunctional replicase when the C_99_-G mutant is used in the assay (J. Pogany and P.D. Nagy, not shown) [31]. Inhibitors DB and GM were added at final concentration of 150 and 100 µM, respectively. After two hours of incubation at room temperature, 1 ml of reaction buffer was added to the *in vitro* assay, followed by incubation on ice for 10 min. Samples were centrifuged at 35,000× g for 1h, and the pellet was washed with 1 ml reaction buffer, followed by centrifugation at 35,000× g for 10 min. The membrane-bound repRNA was extracted from the pellet by adding 0.1 ml stop buffer and 0.1 ml phenol/chloroform and vortexing, followed by isopropanol/ammonium acetate precipitation [52]. The RNA samples were analyzed by denaturing PAGE and phophoimaging as described [52].

## Supporting Information

Figure S1 Schematic presentation of the random mutagenesis strategy used to obtain 6,000 eEF1A mutants. (A) The yeast strain (*tef1*Δ*tef2*Δ carried a plasmid that expressed one of the random eEF1A (*TEF1*) mutants from the native promoter. Each yeast strain also carried pESCHIS4-ADH-His33/CUP1-DI-72 and pRS317-Tet-His92 to induce TBSV repRNA replication as described in the M&M section. (B) Additional experiments on the effect of eEF1A mutations on TBSV repRNA accumulation in yeast. The yeast strain expressed only one form of eEF1A, as indicated. Top panel: Replication of the TBSV repRNA was measured by Northern blotting 24 h after initiation of TBSV replication. (C) The accumulation level of repRNA was normalized based on the rRNA (the 18S ribosomal RNA levels were estimated by Northern blotting). Panels (D), (E) and (F) show the accumulation of p92^pol^, p33 and eEF1A, respectively, estimated by Western blotting using anti-His and anti-eEF1A antibody. (G) SDS-PAGE analysis of total protein extract from the above yeast strains, after Coomassie blue-staining.
(0.15 MB PDF)

Figure S2 Binding of eEF1A to TBSV and TCV replication proteins *in vitro*. (A) MBP-tagged TCV p88C (lacking the p28-overlapping domain from the N-terminus), MBP-TBSV p92, MBP-TBSV p92C (lacking the p33-overlapping domain from the N-terminus) and MBP-TBSV p33 or MBP (1 µg each) were separately immobilized on amylose beads, followed by incubation with a cytosolic extract prepared from yeast. The bound host proteins were eluted from the beads and were analyzed by 10% SDS-PAGE and detected via Western blotting using anti-eEF1A antibody (Top panel). The affinity-purified recombinant MBP-TCV p88C, MBP-TBSV p92, MBP-TBSV p92C, MBP-TBSV p33 and MBP were analyzed by 10% SDS-PAGE and Coomassie blue-staining (Bottom panel). (B) The effect of eEF1A mutations on binding to the viral p33 and p92 proteins *in vitro*. MBP-tagged p92, p33 or MBP were separately immobilized on amylose beads, followed by incubation with a cytosolic extract prepared from yeast expressing wt or mutated eEF1A. The bound eEF1A was eluted from the beads and were analyzed by 10% SDS-PAGE and detected via Western blotting using anti-eEF1A antibody (Top panel). The affinity-purified recombinant MBP-TBSV p92, MBP-TBSV p33 and MBP were analyzed by 10% SDS-PAGE and Coomassie blue-staining (Bottom panel). (C) The effect of eEF1A mutations on binding to the viral repRNA. CFE containing WT or mutated eEF1A was incubated with biotin-labeled DI-72(+) repRNA. Then the repRNA was captured with streptavidin-coated magnetic beads, followed by elution of the co-purified proteins from the beads. Western blot analysis shows the amount of co-purified eEF1A using anti-eEF1A antibody.
(0.12 MB PDF)

Figure S3 Lack of inhibition of TBSV repRNA replication by Cyclohexamide in a cell-free TBSV replicase assay. The cell-free TBSV replicase assay was performed as described in Fig. 4. Cyclohexamide was added in the following amounts: 0, 2, 10, 50, 100 µg/µl.
(0.05 MB PDF)

Figure S4 Lack of inhibition of the *in vitro* activity of the purified tombusvirus replicase by Didemnin B and Gamendazole. (A) The membrane-bound tombusvirus replicase in a yeast lysate was solubilized with Triton X-100/SB3-10 detergent, followed by purification on a FLAG-affinity column as described. The activity of the affinity-purified TBSV replicase was tested on the same amount of DI-72(−) RNA added to each sample. DB (panel on the left) and GM (panel on the right) were added in the following amounts: 0, 100, 150, 200, 250 µM for DB and 0, 25, 50, 100, 200 µM for GM. Denaturing PAGE analysis of the ^32^P-labeled RNA products obtained with the purified tombusvirus replicase is shown. Note that this replicase preparation is only capable of complementary RNA synthesis on the added template RNA, but incapable of supporting a full cycle of replication. (B) The effect of GM and DB on binding to the viral p33 and p92 proteins *in vitro*. MBP-tagged p92 and p33 were separately immobilized on amylose beads, followed by incubation in the presence of 0 or 100 µM GM or 150 µM DB with a cytosolic extract prepared from yeast expressing wt eEF1A. The bound eEF1A was eluted from the beads and were analyzed by 10% SDS-PAGE and detected via Western blotting using anti-eEF1A antibody (Top panel). The affinity-purified recombinant MBP-TBSV p92, MBP-TBSV p33 and MBP were analyzed by 10% SDS-PAGE and Coomassie blue-staining (Bottom panel).
(0.09 MB PDF)
