# Supplementary material for: Translation Elongation Factor 1A Facilitates the Assembly of the Tombusvirus Replicase and Stimulates Minus-Strand Synthesis
Source: PLoS Pathog. 2010 Nov 4;6(11):e1001175. doi: 10.1371/journal.ppat.1001175 (PMC2973826; doi:10.1371/journal.ppat.1001175)
Supplement: Figure S1 — Schematic presentation of the random mutagenesis strategy used to obtain 6,000 eEF1A mutants. (A) The yeast strain (tef1Δtef2Δ carried a plasmid that expressed one of the random eEF1A (TEF1) mutants from the native promoter. Each yeast strain also carried pESCHIS4-ADH-His33/CUP1-DI-72 and pRS317-Tet-His92 to induce TBSV repRNA replication as described in the M&M section. (B) Additional experiments on the effect of eEF1A mutations on TBSV repRNA accumulation in yeast. The yeast strain expressed only one form of eEF1A, as indicated. Top panel: Replication of the TBSV repRNA was measured by Northern blotting 24 h after initiation of TBSV replication. (C) The accumulation level of repRNA was normalized based on the rRNA (the 18S ribosomal RNA levels were estimated by Northern blotting). Panels (D), (E) and (F) show the accumulation of p92pol, p33 and eEF1A, respectively, estimated by Western blotting using anti-His and anti-eEF1A antibody. (G) SDS-PAGE analysis of total protein extract from the above yeast strains, after Coomassie blue-staining. (0.15 MB PDF) [file ppat.1001175.s001.pdf]

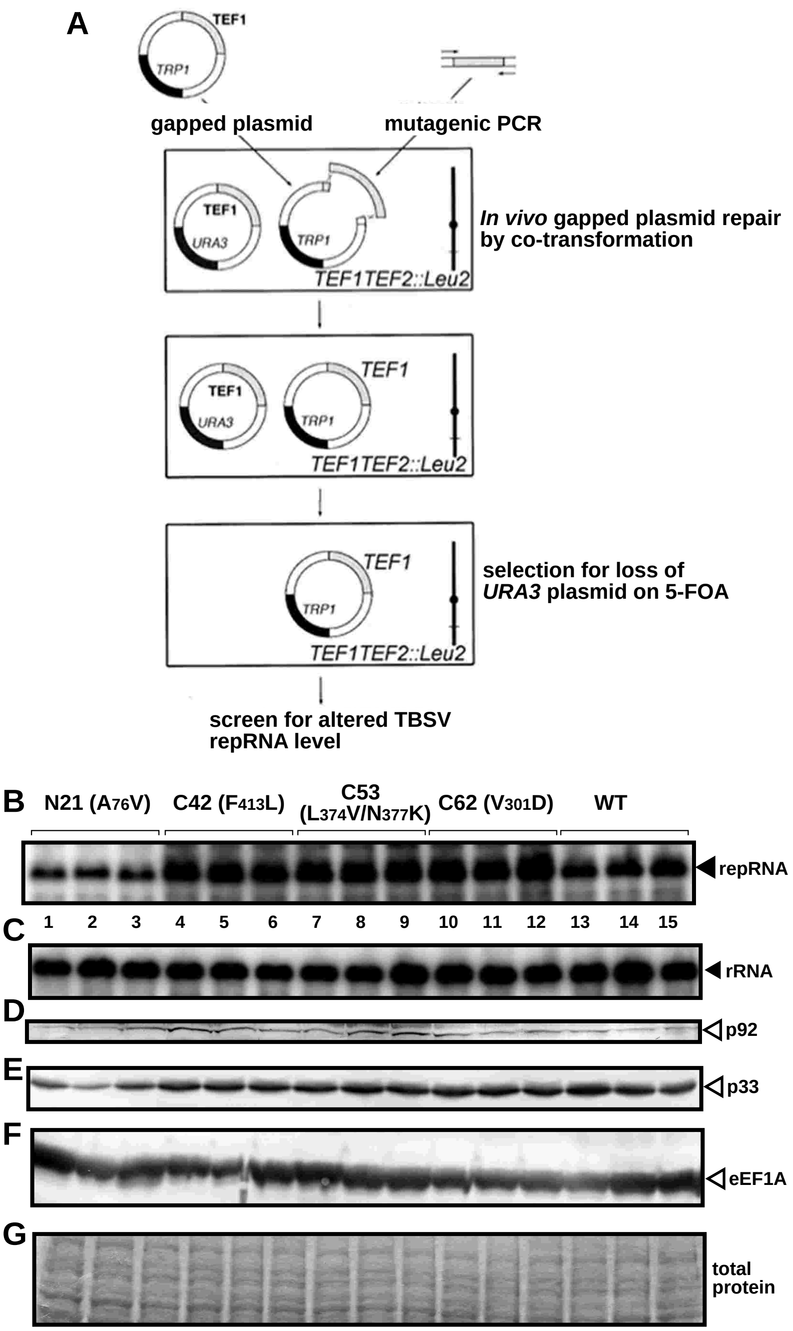
A
TEF1
TRP1
gapped plasmid
mutagenic PCR
TEF1
URA3
TRP1
TEF1TEF2::Leu2
In vivo gapped plasmid repair by co-transformation
TEF1
URA3
TEF1
TRP1
TEF1TEF2::Leu2
TEF1
TRP1
TEF1TEF2::Leu2
selection for loss of URA3 plasmid on 5-FOA
screen for altered TBSV repRNA level
B
N21 (A76V)
C42 (F413L)
C53 (L374V/N377K)
C62 (V301D)
WT
repRNA
1 2 3 4 5 6 7 8 9 10 11 12 13 14 15
C
rRNA
D
p92
E
p33
F
eEF1A
G
total protein

Fig. S1. (A) Schematic presentation of the random mutagenesis strategy used to obtain 6,000 *tef1Δ tef2Δ* yeast strains carrying a plasmid that expressed mutant eEF1A (*TEF1*) from the native promoter. Each yeast strain also carried pESCHIS4-ADH-His33/CUP1-DI-72 and pRS317-Tet-His92 to induce TBSV repRNA replication as described in the M&M section. (B) Additional experiments on the effect of eEF1A mutations on TBSV repRNA accumulation in yeast. The yeast strain expressed only one form of eEF1A, as indicated. Top panel: Replication of the TBSV repRNA was measured by Northern blotting 24 h after initiation of TBSV replication. (C) The accumulation level of repRNA was normalized based on the rRNA (the 18S ribosomal RNA levels were estimated by Northern blotting). Panels (D), (E) and (F) show the accumulation of $p92^{pol}$, p33 and eEF1A, respectively, estimated by Western blotting using anti-His and anti-eEF1A antibody. (G) SDS-PAGE analysis of total protein extract from the above yeast strains, after Coomassie blue-staining.
