# Supplementary material for: Translation Elongation Factor 1A Facilitates the Assembly of the Tombusvirus Replicase and Stimulates Minus-Strand Synthesis
Source: PLoS Pathog. 2010 Nov 4;6(11):e1001175. doi: 10.1371/journal.ppat.1001175 (PMC2973826; doi:10.1371/journal.ppat.1001175)
Supplement: Figure S2 — Binding of eEF1A to TBSV and TCV replication proteins in vitro. (A) MBP-tagged TCV p88C (lacking the p28-overlapping domain from the N-terminus), MBP-TBSV p92, MBP-TBSV p92C (lacking the p33-overlapping domain from the N-terminus) and MBP-TBSV p33 or MBP (1 µg each) were separately immobilized on amylose beads, followed by incubation with a cytosolic extract prepared from yeast. The bound host proteins were eluted from the beads and were analyzed by 10% SDS-PAGE and detected via Western blotting using anti-eEF1A antibody (Top panel). The affinity-purified recombinant MBP-TCV p88C, MBP-TBSV p92, MBP-TBSV p92C, MBP-TBSV p33 and MBP were analyzed by 10% SDS-PAGE and Coomassie blue-staining (Bottom panel). (B) The effect of eEF1A mutations on binding to the viral p33 and p92 proteins in vitro. MBP-tagged p92, p33 or MBP were separately immobilized on amylose beads, followed by incubation with a cytosolic extract prepared from yeast expressing wt or mutated eEF1A. The bound eEF1A was eluted from the beads and were analyzed by 10% SDS-PAGE and detected via Western blotting using anti-eEF1A antibody (Top panel). The affinity-purified recombinant MBP-TBSV p92, MBP-TBSV p33 and MBP were analyzed by 10% SDS-PAGE and Coomassie blue-staining (Bottom panel). (C) The effect of eEF1A mutations on binding to the viral repRNA. CFE containing WT or mutated eEF1A was incubated with biotin-labeled DI-72(+) repRNA. Then the repRNA was captured with streptavidin-coated magnetic beads, followed by elution of the co-purified proteins from the beads. Western blot analysis shows the amount of co-purified eEF1A using anti-eEF1A antibody. (0.12 MB PDF) [file ppat.1001175.s002.pdf]

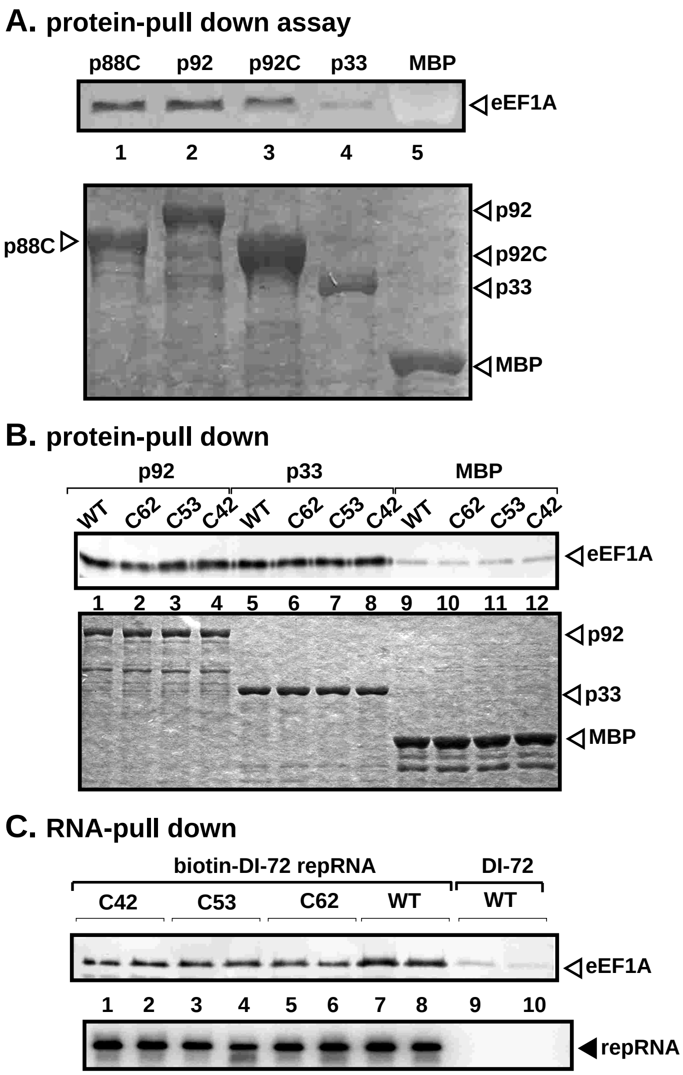
A. protein-pull down assay
p88C
p92
p92C
p33
MBP
eEF1A
1 2 3 4 5
p88C
p92
p92C
p33
MBP
B. protein-pull down
p92
p33
MBP
WT C62 C53 C42 WT C62 C53 C42 WT C62 C53 C42
eEF1A
1 2 3 4 5 6 7 8 9 10 11 12
p92
p33
MBP
C. RNA-pull down
biotin-DI-72 repRNA
DI-72
C42
C53
C62
WT
WT
eEF1A
1 2 3 4 5 6 7 8 9 10
repRNA

Fig. S2. (A) Binding of eEF1A to TBSV and TCV replication proteins *in vitro*. MBP-tagged TCV p88C (lacking the p28-overlapping domain from the N-terminus), MBP-TBSV p92, MBP-TBSV p92C (lacking the p33-overlapping domain from the N-terminus) and MBP-TBSV p33 or MBP (1 μg each) were separately immobilized on amylose beads, followed by incubation with a cytosolic extract prepared from yeast. The bound host proteins were eluted from the beads and were analyzed by 10% SDS-PAGE and detected via Western blotting using anti-eEF1A antibody (Top panel). The affinity-purified recombinant MBP-TCV p88C, MBP-TBSV p92, MBP-TBSV p92C, MBP-TBSV p33 and MBP were analyzed by 10% SDS-PAGE and Coomassie blue-staining (Bottom panel). (B) The effect of eEF1A mutations on binding to the viral p33 and p92 proteins *in vitro*. MBP-tagged p92, p33 or MBP were separately immobilized on amylose beads, followed by incubation with a cytosolic extract prepared from yeast expressing wt or mutated eEF1A. The bound eEF1A was eluted from the beads and were analyzed by 10% SDS-PAGE and detected via Western blotting using anti-eEF1A antibody (Top panel). The affinity-purified recombinant MBP-TBSV p92, MBP-TBSV p33 and MBP were analyzed by 10% SDS-PAGE and Coomassie blue-staining (Bottom panel). (C) The effect of eEF1A mutations on binding to the viral repRNA. CFE containing WT or mutated eEF1A was incubated with biotin-labeled DI-72(+) repRNA. Then the repRNA was captured with streptavidin-coated magnetic beads, followed by elution of the co-purified proteins from the beads. Western blot analysis shows the amount of co-purified eEF1A using anti-eEF1A antibody.
