# Supplementary material for: Translation Elongation Factor 1A Facilitates the Assembly of the Tombusvirus Replicase and Stimulates Minus-Strand Synthesis
Source: PLoS Pathog. 2010 Nov 4;6(11):e1001175. doi: 10.1371/journal.ppat.1001175 (PMC2973826; doi:10.1371/journal.ppat.1001175)
Supplement: Figure S3 — Lack of inhibition of TBSV repRNA replication by Cyclohexamide in a cell-free TBSV replicase assay. The cell-free TBSV replicase assay was performed as described in Fig. 4. Cyclohexamide was added in the following amounts: 0, 2, 10, 50, 100 µg/µl. (0.05 MB PDF) [file ppat.1001175.s003.pdf]

Cell-free replicase assay:

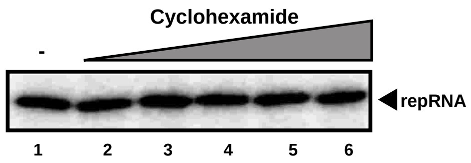

Fig. S3. Lack of inhibition of TBSV repRNA replication by Cyclohexamide in a cell-free TBSV replicase assay. The cell-free TBSV replicase assay was performed as described in Fig. 4. Cyclohexamide was added in the following amounts: 0, 2, 10, 50, 100 μg/μl.
