# Supplementary material for: Translation Elongation Factor 1A Facilitates the Assembly of the Tombusvirus Replicase and Stimulates Minus-Strand Synthesis
Source: PLoS Pathog. 2010 Nov 4;6(11):e1001175. doi: 10.1371/journal.ppat.1001175 (PMC2973826; doi:10.1371/journal.ppat.1001175)
Supplement: Figure S4 — Lack of inhibition of the in vitro activity of the purified tombusvirus replicase by Didemnin B and Gamendazole. (A) The membrane-bound tombusvirus replicase in a yeast lysate was solubilized with Triton X-100/SB3-10 detergent, followed by purification on a FLAG-affinity column as described. The activity of the affinity-purified TBSV replicase was tested on the same amount of DI-72(−) RNA added to each sample. DB (panel on the left) and GM (panel on the right) were added in the following amounts: 0, 100, 150, 200, 250 µM for DB and 0, 25, 50, 100, 200 µM for GM. Denaturing PAGE analysis of the 32P-labeled RNA products obtained with the purified tombusvirus replicase is shown. Note that this replicase preparation is only capable of complementary RNA synthesis on the added template RNA, but incapable of supporting a full cycle of replication. (B) The effect of GM and DB on binding to the viral p33 and p92 proteins in vitro. MBP-tagged p92 and p33 were separately immobilized on amylose beads, followed by incubation in the presence of 0 or 100 µM GM or 150 µM DB with a cytosolic extract prepared from yeast expressing wt eEF1A. The bound eEF1A was eluted from the beads and were analyzed by 10% SDS-PAGE and detected via Western blotting using anti-eEF1A antibody (Top panel). The affinity-purified recombinant MBP-TBSV p92, MBP-TBSV p33 and MBP were analyzed by 10% SDS-PAGE and Coomassie blue-staining (Bottom panel). (0.09 MB PDF) [file ppat.1001175.s004.pdf]

A. In vitro replicase assay:

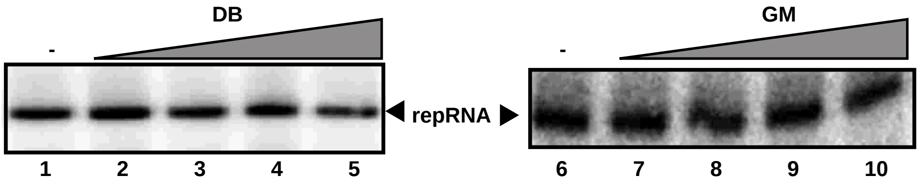

B. protein-pull down

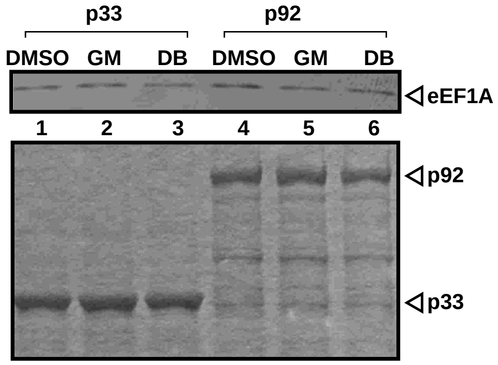

Fig. S4. (A) Lack of inhibition of the *in vitro* activity of the purified tombusvirus replicase by Didemnin B and Gamendazole. The membrane-bound tombusvirus replicase in a yeast lysate was solubilized with Triton X-100/SB3-10 detergent, followed by purification on a FLAG-affinity column as described . The activity of the affinity-purified TBSV replicase was tested on the same amount of DI-72(-) RNA added to each sample. DB (panel on the left) and GM (panel on the right) were added in the following amounts: 0, 100, 150, 200, 250 μM for DB and 0, 25, 50, 100, 200 μM for GM. Denaturing PAGE analysis of the ^{32}P-labeled RNA products obtained with the purified tombusvirus replicase is shown. Note that this replicase preparation is only capable of complementary RNA synthesis on the added template RNA, but incapable of supporting a full cycle of replication. (B) The effect of GM and DB on binding to the viral p33 and p92 proteins *in vitro*. MBP-tagged p92 and p33 were separately immobilized on amylose beads, followed by incubation in the presence of 0 or 100 μM GM or 150 μM DB with a cytosolic extract prepared from yeast expressing wt eEF1A. The bound eEF1A was eluted from the beads and were analyzed by 10% SDS-PAGE and detected via Western blotting using anti-eEF1A antibody (Top panel). The affinity-purified recombinant MBP-TBSV p92, MBP-TBSV p33 and MBP were analyzed by 10% SDS-PAGE and Coomassie blue-staining (Bottom panel).
